# The VOICE Study: Valuing Opinions, Individual Communication and Experience: building the evidence base for undertaking Patient-Centred Family Meetings in palliative care - a mixed methods study

**DOI:** 10.1186/s40814-017-0225-9

**Published:** 2018-02-20

**Authors:** Philippa J. Cahill, Christine R. Sanderson, Elizabeth A. Lobb, Jane L. Phillips

**Affiliations:** 1 School of Medicine, University of Notre Dame Australia, Darlinghurst, Australia; 2 Faculty of Health, University of Technology Sydney, Ultimo, Australia; 3 School of Nursing, University of Notre Dame Australia, Darlinghurst, Australia; 4 Calvary Health Care Kogarah, Kogarah, Australia; 5 School of Medicine, University of Notre Dame Australia, 160 Oxford Street, Darlinghurst, NSW 2010 Australia

**Keywords:** Palliative care, Family, Meeting, Conference, Terminal care

## Abstract

**Background:**

Despite family meetings being widely used to facilitate discussion among patients, families, and clinicians in palliative care, there is limited evidence to support their use. This study aims to assess the acceptability and feasibility of Patient-Centred Family Meetings in specialist inpatient palliative care units for patients, families, and clinicians and determine the suitability and feasibility of validated outcome measures from the patient and family perspectives.

**Methods:**

The study is a mixed-methods quasi-experimental design with pre-planned Patient-Centred Family Meetings at the intervention site. The patient will set the meeting agenda a priori allowing an opportunity for their issues to be prioritised and addressed. At the control site, usual care will be maintained which may include a family meeting. Each site will recruit 20 dyads comprising a terminally ill inpatient and their nominated family member. Pre- and post-test administration of the Distress Thermometer, QUAL-EC, QUAL-E, and Patient Health Questionnaire-4 will assess patient and family distress and satisfaction with quality of life. Patient, family, and clinician interviews post-meeting will provide insights into the meeting feasibility and outcome measures. Recruitment percentages and outcome measure completion will also inform feasibility.

Descriptive statistics will summarise pre- and post-meeting data generated by the outcome measures. SPSS will analyse the quantitative data. Grounded theory will guide the qualitative data analysis.

**Discussion:**

This study will determine whether planned Patient-Centred Family Meetings are feasible and acceptable and assess the suitability and feasibility of the outcome measures. It will inform a future phase III randomised controlled trial.

**Trial registration:**

Australian New Zealand Clinical Trials Registry ACTRN12616001083482 on 11 August 2016

## Background

Effective communication in palliative care practice is central to adequately addressing patient and family needs and concerns [1–3]. The bringing together of patients, family, and clinicians for a purposeful discussion has been recognised as a means of assisting with patient-family and clinical team communication in the specialist palliative care setting [4–7]. This “family meeting” or “family conference,” as it is variously referred to in the literature [5, 6], facilitates discussion about the illness experience, care choices, and end-of-life decision-making [5, 8, 9]. In this setting, “family” is defined as whomever the patient designates as “family” and may include close friends or carers [10].

Only a limited number of studies have been undertaken to examine the outcomes of family meetings. Hannon et al. [11] demonstrated that unmet family needs and concerns were significantly improved as a result of family meetings. The authors recommended further studies to review the impact of family meetings from the family and clinician perspective and to incorporate qualitative data collection.

A prospective study [12] of family meetings demonstrated that the high levels of verbal expression of distress exhibited by primary caregivers were not significantly affected by the presence of the patient. However, other family members verbally expressed their emotional distress more often when the patient was absent. Patient presence was also significantly associated with increased discussion regarding goals of care and decreased discussion about prognosis and the symptoms dying patients may experience [12]. In this study, no validated measures were used to assess distress, and patients did not routinely participate in the family meetings. The authors of the study [12] recommended identification of patient preferences for family meeting structure and procedures, and that consideration should be given to a randomised control trial (RCT) to compare patient and family participation with family only participation in family meetings.

*Guidelines for Conducting Family Meetings in Palliative Care* were developed in Australia [6], and provide a structured approach for preparing and conducting a family meeting. An evaluation of the Guidelines’ effectiveness [13] and their impact on family outcomes demonstrated a significant increase post-meeting in the overall means for addressing important family needs [13]. The timing of the family meeting and a review of patient benefits were suggested as important areas for further research [13]. The authors concluded that family meetings were acceptable and feasible to ascertain and deal with family concerns; however, the authors did not document the feasibility and acceptability for patients and clinicians.

A recent systematic review [14] examined what measureable health outcomes improved as a result of family meetings convened in acute and sub-acute inpatient care settings, including palliative care [14]. This review found low-to-moderate positive evidence that family meetings led to a reduction in family psychological distress and addressed the information and support needs of families [14]. Further research to assess patient outcomes and the patient’s role in family meetings was recommended. A more recent review found a dearth of evidence to support the use of family meetings in palliative care [15]. In particular, there was no study that demonstrated patient benefits using a validated measure and only two studies used a validated measure to demonstrate family benefits as a result of participation in a family meeting. There was also limited qualitative evidence to support positive outcomes for patients, families and clinicians participating in a family meeting.

Overall there is limited high-level evidence to demonstrate the benefits of family meetings for patients, families and clinicians in the palliative care context. The feasibility and acceptability of family meetings has only been confirmed for families [13]. The authors of published studies have recommended that additional research is required to assess patient outcomes, preferences, and participation in family meetings in the palliative care setting [12, 13] and that clinician outcomes should also be evaluated [11]. The use of qualitative data [11], to assess the impact of family meetings on family participants and to examine the timing of family meetings [13] have also been advocated as areas for further investigation. In addition, the majority of studies have not used validated measures to evaluate the impact of family meetings on participants, in particular patient and family distress [12, 14, 16].

Therefore, there is a clear need to undertake research using quantitative and qualitative data to assess the feasibility, acceptability and impact of family meetings from the perspective of all participants - patient, family and clinicians. A clearer understanding of the patient’s role and their preferences for family meetings is imperative given: (a) the vulnerability of this patient cohort; (b) the uncertainty regarding the value of family meetings in palliative care; (c) the resource intensity of clinician time associated with this intervention [11]; and (d) the current intuitive belief that these meetings are an important aspect of standard care [8, 17].

In the proposed study, qualitative data collection with interviews of patients, families and clinician focus groups will assess the feasibility and acceptability of Patient-Centred Family Meetings and the suitability and feasibility of the measures used. Patients will complete the QUAL-EC questionnaire [18] and family members will complete the corresponding QUAL-E (Family) questionnaire [19] to measure patient and family satisfaction with quality of life at end-of-life. Patients and families will complete the National Cancer Collaborative Network (NCCN) Distress Thermometer [20] as a measure of distress and families will complete the PHQ-4 (Patient Health Questionnaire-4) [21–23] as an additional distress measure.

As recruitment will be from a palliative care population, patients who are terminally ill or debilitated can affect data collection. This issue will be addressed by the eligibility criteria. The Palliative Care Outcomes Collaboration (PCOC) palliative care phase classification system [24] will be used to confirm that the patient is not in the terminal phase and the Australian Modified Karnofsky Performance Score [25] will be used to confirm the physical capability of the patient to participate in the VOICE Study.

### Objectives

The primary objectives of the VOICE Study are to:

(i) Assess the acceptability and feasibility of providing planned Patient-Centred Family Meetings in a specialist inpatient palliative care unit for patients, families and clinicians;
(ii) Understand the benefits and burden of participating in a Patient-Centred Family Meetings from the patient, family and clinician perspective; and
(iii) Assess the suitability and feasibility of the selected outcome measures for patients and families.

The secondary objective is to inform the development of a future phase III RCT.

The principles of patient-centred care [26] will underpin the proposed intervention and are congruent with the clinical practice of palliative care [27]. For this reason, the family meeting intervention in this study will be referred to as a Patient-Centred Family Meeting. The recognition of the patient as an individual and the obligation to acknowledge their unique needs, wants, values and concerns and those of their family and/or significant others (if present) is paramount. The Patient-Centred Family Meeting will focus on the patient and give them a “voice” by facilitating the meeting discussion concerning their needs, concerns opinions and experiences. The VOICE acronym reflects this concept. By also assessing the acceptability and feasibility of Patient-Centred Family Meetings from the perspective of families and clinicians, all participants are given a “voice” in the VOICE Study.

### Research design

The VOICE Study Research Protocol (Version 5.6, 11.3.2017) will utilise a mixed methods quasi-experimental (pre-post-test) design. The specific strategy used to mitigate factors that may compromise internal validity will be a non-equivalent control group design. This design incorporates a comparison (control) group and collection of equivalent pre-test and post-test measures for the intervention and comparison (control) groups [28]. The SPIRIT checklist [29] was used to inform the study protocol and is included as an additional file.

## Methods/Design

### Study setting

Two sub-acute facilities in Sydney, Australia who provide specialist inpatient and community palliative care to the local geographic population will be included in the VOICE Study with one being a control site and the other the intervention site. The study design will incorporate four study groups:

(i) Inpatients receiving the intervention of a planned Patient-Centred Family Meeting;
(ii) Family member(s) (or equivalent person) who are invited by the patient to participate in a Patient-Centred Family Meeting;
(iii) Inpatients receiving standard care (who may also participate in a “standard” family meeting);
(iv) Family member(s) (or equivalent person) nominated by the patient to participate in the VOICE Study.

The control site will provide their usual palliative care, which may or may not include a family meeting. This control site will provide insights into standard palliative care practices, such as how the team determines who needs a family meeting. The intervention site will provide a planned Patient-Centred Family Meeting for patients and families. The same outcome measures will be collected at each site to help identify factors or confounding variables that operate in standard palliative care practice that may not be apparent at the intervention site. For the purposes of this research study, “family” is defined as whomever the patient designates as “family” and may include close friends or carers or significant others [10].

At each facility, the Director of the Palliative Care Service will act as the on-site Principal Investigator and will liaise with the on-site palliative care clinical staff and the PhD student (corresponding author). The other authors will be supervising the PhD student for the VOICE Study and will be involved in decisions concerning data management and publications and consultation with the on-site principal investigators as required.

### Participant eligibility criteria

#### Inclusion criteria

##### Patients

All patients (aged ≥ 18 years) admitted to a specialist palliative care unit within the last 7 days with a terminal illness (a prognosis of ≤ 12 months, but expected to live at least 14 days, i.e., the average length of the protocol) will be included. The patient must be able to identify a primary support person who agrees to participate in the Patient-Centred Family Meeting (intervention arm) or the control arm of the study. In order to be well enough to participate in a Patient-Centred Family Meeting, the patient will have a Australian Modified Karnofsky Performance Score ≥ 30 [25]. The patient must also be classified at recruitment as being either “Stable”, “Unstable,” or “Deteriorating” in accordance with Palliative Care Outcomes Collaboration (PCOC) palliative care phase classification system [24].

##### Family

Participating family members must be aged ≥ 18 years.

All patient and family participants will have sufficient English language and cognitive skills to complete an informed consent, complete baseline information and questionnaires and contribute effectively in the Patient-Centred Family Meeting or family meeting if it occurs as part of usual care. The researchers recognise the importance of non-English speaking patients and families in the provision of quality palliative care and providing them with the opportunity to participate in research studies. However, the validated palliative care measures which will be used in this study have not been validated for patients with insufficient English literacy to complete the questionnaires [18].

#### Exclusion criteria

Patients who are receiving treatment for curative intent, who are in the “Terminal” phase as measured by PCOC assessment [24] or who are cognitively impaired, will be excluded.

Family members who have not been invited to attend the Patient-Centred Family Meeting (intervention) by a participating patient will be excluded.

### Intervention

The intervention is a planned face-to-face Patient-Centred Family Meeting for the inpatient and family member(s) after inpatient admission to a specialist palliative care facility that is the designated intervention site. The duration of the Meeting will be 60 min, although this may vary depending on patient and family members’ needs. The intervention is designed to be “patient-centred” with the patient setting the agenda in advance so that their concerns and issues are addressed. The baseline information about the patient's issues, concerns and expectations of their inpatient stay will inform the Patient-Centred Family Meeting agenda. Meeting discussion may include clinical and psycho-social issues, preparation for death and other key areas of importance for that patient and family. The Meeting will be scheduled as soon as practicable after the patient’s admission and be conducted in a private space with members of the palliative care multidisciplinary team. Routinely, the palliative care consultant and/or registrar and social worker will attend. Other members of the team will participate as required or as requested by the patient during preparation for the meeting. A participant attendance sheet will be completed.

The palliative care consultant or social worker will facilitate the meeting, according to the Manual for Patient-Centred Family Meetings. The researcher will provide education to the clinical staff prior to commencement of the study and will attend Patient-Centred Family Meetings to observe for fidelity to the Manual procedures.

Other than the Patient-Centred Family Meeting, all other usual palliative care practices will be provided to the patient and their family.

### Control

The Patient-Centred Family Meeting will not be offered at the control site. Usual care will be maintained which may include a family meeting that is held as part of the facility’s usual care. The need for a family meeting will be determined as per the facility’s usual clinical practice. A proportion of these “standard” family meetings will be observed using the family meeting observation sheet.

For both the intervention and control sites, if, following consent, the patient’s mental state changes, the researcher or clinical trials nurse will consult the clinical team concerning the appropriateness of the patient continuing in the study. The clinical team will be asked to consider the patient’s mental state to assess if the patient fulfils the exclusion criteria of cognitive impairment. If the patient does fulfil the exclusion criteria, the patient will be withdrawn from the study. The family member(s) participating in the study will be informed and the family member will also be withdrawn from the study.

### Outcomes

Both qualitative and quantitative data and will be collected for the VOICE Study. Qualitative data will assess the primary outcome of the VOICE Study to determine the feasibility and acceptability of a Patient-Centred Family Meeting in specialist palliative care units for all participants. This data will also contribute to understanding the benefits and burden of participating in a Patient-Centred Family Meeting from the perspective of all participants. Qualitative and quantitative data will also be used to determine the secondary outcome of the VOICE Study concerning the suitability and feasibility of the proposed outcome measures for patients and families.

#### Qualitative data

At the intervention site, a Patient-Centred Family Meeting patient semi-structured interview schedule will be undertaken with participating patients 1–2 days post-meeting that includes questions related to the feasibility and acceptability of the Patient-Centred Family Meeting such as: “What was the family meeting like for you?” This interview will also be undertaken at the control site using the same interview schedule if the patient participates in a family meeting as part of standard care. At both sites, patients will be asked about the measures used to assess their suitability and feasibility.

At the intervention site, the family participant will complete a Patient-Centred Family Meeting feedback questionnaire post meeting. At the control site, a family meeting feedback questionnaire will be completed post meeting if a family participates in a family meeting as part of standard care. Both feedback questionnaires will include questions about the family’s experience of the positive and negative aspects of the meeting attended. Family semi-structured interviews will also be undertaken at both sites to discuss questions related the feasibility and acceptability of the Patient-Centred Family Meeting or "standard" family meeting for example “Do you think the family meeting was helpful or not helpful for the family members who attended?” and to also assess the suitability and feasibility of the measures used. All interviews will follow a semi-structured schedule to ensure consistency.

At the intervention site, the Patient-Centred Family Meeting multidisciplinary staff focus group semi-structured interview schedule will be used to evaluate the feasibility and acceptability of clinicians participating in Patient-Centred Family Meetings. Focus groups will use a moderator and an observer to ensure consistency across each focus group and keep the participants on track. Questions such as “Do you think it is feasible to provide these meetings as part of standard care?” will be asked. The clinician focus groups will include members of the multidisciplinary specialist palliative care team e.g. medical, nursing and allied health clinicians who have been involved in the care of patients and their families, and participated in at least one patient-centred family meeting. The purpose of these focus groups is to (a) explore the clinicians’ experience of the Patient-Centred Family Meetings process; (b) identify positive and less positive aspects; and (c) assess the acceptability and feasibility of the model such as the time involved and the benefits. The clinician focus groups provide a “voice” for the multidisciplinary team members and echo the VOICE Study acronym.

A family meeting observation sheet will record key elements of the Patient-Centred Family Meeting during the meeting. The data will include who attended the meeting, reasons for participants’ non-attendance and record which components of the pre-set patient agenda were discussed.

#### Quantitative measures

Measures of patient and family satisfaction with quality of life at end-of-life and patient and family distress will be collected using validated measures, and these measures will be evaluated qualitatively as described above, to assess their suitability and feasibility for patients and families.

The QUAL-EC [18] questionnaire will assess the consented patients’ symptom control, their relationship with the healthcare provider, life completion, and preparation for end-of-life. The QUAL-E (Family) [19] questionnaire will measure the experience of consented family members’ whose relatives are enrolled in the VOICE Study. The questionnaire will assess the relationship with the healthcare provider and the completion of the relationship with the patient. The family will also report on the patient’s symptom experience, preparation for death, and state of peace.

The NCCN Distress Thermometer [20] will be used to assess changes in distress of patients and family members pre- and post-patient-centred family meetings. The PHQ-4 [21–23] combines the Patient Health Questionnaire-2 and Generalised Anxiety Disorder (GAD)-2, and will be used as an additional distress measure for families.

A single question measure: “To what extent are you at peace?” will explore the patients’ spiritual and emotional well-being [30].

The use of the QUAL-EC as a patient outcome measure is based on the rationale that the quality of life at the end of life measures and domains assessed may be impacted by participation in the Patient-Centred Family Meeting. The rationale for the use of the QUAL-E (Family) tool was based on it being the companion instrument for the QUAL-EC and that it will also measure domains of the family experience that may be affected by participation in a Patient-Centred Family Meeting.

The NCCN Distress Thermometer instrument has been used previously to assess changes in distress of key family members pre- and post-family meetings [31]. The VOICE Study will provide an opportunity to assess the feasibility and suitability of the PHQ-4 tool in the context of palliative care and is not likely to be burdensome for the family member. However as it was considered an additional burden for the patient it will not be used. In the event that the scores obtained on either the NCCN Distress Thermometer or PHQ-4 indicate significant distress, the clinical team will be informed.

The peace question will be used in the VOICE Study as an additional patient measure to assess any differences in the patients’ perception of peace pre and post participation in the Patient-Centred Family Meeting.

### Sample size

Twenty inpatients will be recruited from each facility. At each site, the patient will nominate one family member (or equivalent) as the primary support person to participate in the study. A total of 20 patient-family dyads will be recruited for each site, resulting in 40 participants at each site and a total of 80 participants for the entire study. The total number may exceed this if additional family participants wish to attend the Patient-Centred Family Meeting or standard care family meeting. Data will be collected from additional family participants who consent to study inclusion. A power calculation was not undertaken as this will be a feasibility study.

### Recruitment

The researcher at the intervention site and the clinical trials nurses at the control site will review the clinical record of newly admitted patients and undertake necessary screening for eligibility, and if required, speak with the clinical staff to clarify any aspects of inclusion criteria such as cognitive status. This visit will not directly involve the patient. During the course of the study at the intervention site, the researcher will maintain a visible presence at the facility to ensure that all eligible patients are identified and recruited in a timely manner. The involvement of experienced clinical trials nurses at the control site is expected to enhance participant recruitment to the study at this facility.

### Data collection methods for the intervention arm of the study

The patient and family participant timeline and data collection for both arms of the study are summarised in Table 1, and a detailed description of these key tasks for the intervention arm is outlined below.

**Table 1:**
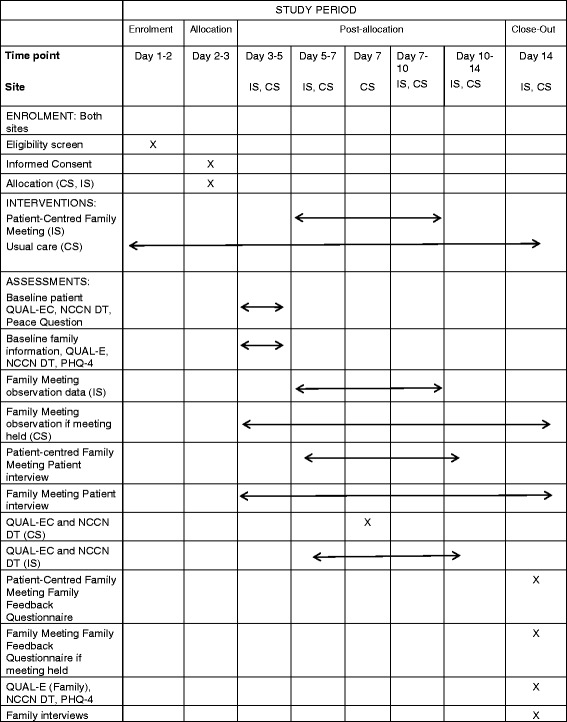
VOICE Study patient and family participant timeline for enrolment, interventions, and assessments at intervention and control sites

*Day 1* day of admission, *CS* control site, *IS* intervention site, *NCCN DT* National Cancer Collaborative Network Distress Thermometer, *PHQ-4* Patient Health Question Questionnaire-4

#### Days 1–2 screening

Recruitment of patients and family members will commence Days 1–2 following admission. The researcher will review the clinical record of all newly admitted patients to screen for eligibility according to the inclusion criteria for the VOICE Study. Clinical staff will clarify any issues or information concerning the patient’s status such as their cognitive status or palliative care phase [24] .

#### Days 2–3 recruitment and consent

On Days 2–3 of admission, all eligible patients will receive a formal written “Invitation to attend a Patient-Centred Family Meeting” and if they are interested, the patient will be given the Patient-Centred Family Meeting Introductory Patient Information sheet. The study will be discussed with the patient and issues addressed by the researcher. Interested patients will be provided with the Patient-Centred Family Meeting Participant (patient) Information Statement and Consent Form. Patients who agree to participate will be asked to complete the consent form. If the patient requires more time to consider participation, the researcher will return at a later date to finalise recruitment.

#### Days 3–5 baseline data collection

Consented patients will then be asked to complete the Patient-Centred Family Meeting Patient Information Booklet that includes:

(i) baseline patient information, demographics and questions that will guide the Patient-Centred Family Meeting agenda,
(ii) the QUAL-EC questionnaire [18, 32]
(iii) the NCCN Distress Thermometer [20]
(iv) the single question measure: “To what extent are you at peace?”.

Consented patients will be asked to nominate and provide contact details of the primary support person who the patient would like to participate in the proposed Patient-Centred Family Meeting. If required, the researcher or social worker will assist with contacting the support person and teleconferencing will be made available if required. The patient may also nominate additional family members or significant others to be included in the study and/or attend the Patient-Centred Family Meeting.

The nominated family member(s) will be contacted and given a copy of the Patient-Centred Family Meeting introductory Family Information. The study will be explained to the family member(s) and questions or issues discussed. A Patient-Centred Family Meeting Participant (family) Information Statement and Consent Form will be provided and further questions addressed. Interested family member(s) who are on site will be consented immediately or the consent form will be completed prior to the Patient-Centred Family Meeting commencing. The Patient-Centred Family Meeting Family Information Booklet which includes questions about the patient and pre-meeting measures: QUAL-E (Family) questionnaire; the NCCN Distress Thermometer; and the PHQ-4 will be completed prior to the meeting.

If an invited family member(s) or significant other(s) do not meet the criteria or will not consent, the patient will nominate an alternative participant. If this is not possible, the patient will be excluded.

#### Days 5–10 Patient-Centred Family Meeting and repeat patient measures

The researcher or social worker will arrange the Patient-Centred Family Meeting in consultation with the patient, family, and clinicians between days 5–10. During the Patient-Centred Family Meeting, the researcher will complete the family meeting observation sheet. Within 24–48 h post-meeting, the researcher will interview the patient using the Patient-Centred Family Meeting patient semi-structured interview schedule. The patient will also complete repeat QUAL-EC and NCCN Distress Thermometer measures.

#### Day 14 repeat family measures

On Day 14, family participant(s) will complete the Patient-Centred Family Meeting family feedback questionnaire and repeat measures of the QUAL-E (Family), NCCN Distress Thermometer and PHQ-4 by telephone or on-site according to their availability. A sample of family members (*n* = 10) will also participate in an interview, using the QUAL-E (Family) questions as prompts for further discussion about families’ views concerning the relevance of the measures, time taken to complete, any issues about the measures and their preference(s) for a particular measure. This interview will be conducted by telephone or face-to-face based on availability.

### Data collection for the intervention arm of the study for clinicians

After the completion of a number of Patient-Centred Family Meetings, a focus group will be convened to seek the views of multidisciplinary team members who have cared for the patients recruited to the VOICE Study and attended at least one Patient-Centred Family Meeting. Additional focus groups will occur during the course of the VOICE Study to include additional team members and ensure data saturation. However, each team member will only be required to attend one focus group.

### Data collection methods for the control arm of the research study (Table 1)

#### Days 1–2 screening

A clinical trials nurse will undertake the same eligibility screening procedures as undertaken at the intervention site.

#### Days 2–3 recruitment and consent

On Days 2–3 of admission, eligible patients will receive the introductory patient information and the study will be discussed with the patient and any questions or concerns addressed. Interested patients will receive the Participant (patient) Information Statement and Consent Form and written consent will be obtained. If the patient requires more time to consider, the clinical trials nurse will return to finalise recruitment.

#### Days 3–5 baseline data collection

The consented patient will complete the Patient Information Booklet that includes information about family, the QUAL-EC and the NCCN Distress Thermometer. Consented patients will nominate a primary support person to participate in the study. The patient may also invite additional family members or significant others to be included in the study and/or attend any family meeting held. The nominated family member(s) will be given study information and any questions or issues discussed. Consented family member(s) will complete the Family Information Booklet that includes patient-related questions and the outcome measures used at the intervention site. If the family member is unavailable, the family member will be requested to complete the consent form and Family Information Booklet by Day 7 of the patient’s admission. If the nominated family member(s) does not meet the criteria or will not consent, the patient will nominate an alternative person. If this is not possible, the patient will be excluded.

#### Day 7 repeat patient measures

On Day 7, the patient will complete repeat measures of the QUAL-EC questionnaire and the NCCN Distress Thermometer. The researcher will interview the patient using the family meeting patient semi-structured interview schedule if a family meeting has been held. If the patient participates in a family meeting after Day 7, the researcher will undertake the interview at a convenient time for the patient.

#### Day 14 repeat family measures

On Day 14 participating family member(s) follows the same intervention site procedures concerning repeat measures. If the family member(s) has attended a family meeting, the family member will complete the Family Meeting Family Feedback Questionnaire on-site or by telephone. A sample of family members (*n* = 10) will participate in an interview, using the QUAL-E (Family) questions as per the intervention site procedures.

The researcher will consult regularly with clinicians to promote participant retention and ensure complete follow-up. Incomplete participant data and the reasons for this will be documented. Pre- and post-measures that are not completed when a participant withdraws from the study will be excluded from the data analysis.

### Data management

All patient and family participants in the VOICE Study will be allocated a unique identification (ID) code that will be entered on all questionnaires and linked with all completed interviews. The relevant ID code will form part of the data entry for the qualitative and quantitative data. All data will be entered and stored on a secure computer hard drive that will be backed up daily and be firewall protected. All computers will be password protected. Only the researcher and her supervisor(s) will have access to the master list that links participants to the de-identified data and all data will be stored separately from the register of participants. All VOICE Study documentation will be placed in a locked filing cabinet in a locked office, in accordance with National Health and Medical Research Council (NH&MRC), Australia requirements [33].

### Statistical methods

Quantitative measures and qualitative data collected for the four study groups will be analysed.

#### Quantitative data

Prior to analysis of the quantitative date, all variables will be assessed for accuracy of input, plausibility of means and standard deviations and out-of-range and missing values. The quantitative data will be analysed using Statistical Package for the Social Sciences (SPSS). Descriptive statistics (frequencies, means and medians) will be used to summarise the baseline and follow-up data including demographics, NCCN DT, PHQ-4, QUAL-EC and QUAL-E (Family) measures and quantitative questions on the feedback questionnaires. Differences on these variables [NCCN DT, PHQ-4, QUAL-EC and QUAL-E (Family)] between the intervention group and control group will be assessed using univariate, parametric or non-parametric statistics depending if these outcomes are normally or non-normally distributed.

#### Qualitative data

All qualitative data will be reviewed prior to the commencement of analysis. Patient interviews will be recorded and transcribed verbatim. Data analysis will be conducted using theoretical and procedural direction from grounded theory research utilising the constant comparative method [34]. The transcripts will be uploaded into the software program QSR NVivo. Open, axial, and selective coding will be used to analyse the data [35]. Iterative data analysis will commence after the first interview with all investigators participating using the selected interview transcripts and additional excerpts to identify and agree upon emergent themes. The open-ended responses from the family feedback questionnaires will be analysed using the constant-comparative method. The clinician focus groups will be recorded, transcribed verbatim, and analysed using the constant comparative method. Sufficient numbers of clinicians will participate in focus groups to ensure that data saturation is achieved.

Additional analysis of the percentage of dyads recruited, family meeting observations and feedback from patients, families and clinicians will provide information about the feasibility of the meetings. The percentage of completed questionnaires, patient and family feedback, preferences and issues about the measures will provide information about their feasibility and suitability from the patient and family perspective.

### Monitoring

A data monitoring committee will not be convened as qualitative data will be reviewed and analysed iteratively and involve all the investigators.

### Harms

The Participant Information Statement and Consent Form for patients and families will indicate that there is small risk that completing the questionnaires and interviews may cause these participants to reflect on their life and situation and become distressed. The form also notes that sharing information about end-of-life issues between patients and family members and hearing of their concerns may also be distressing. The patients and family will be advised that if they become upset or distressed as a result of participation in the VOICE Study, the researcher will inform the treating doctor and the clinical team. In the case of the patient, the clinical team will arrange for counselling or other appropriate support. For family members, the clinical team will provide information about support options available if that is their wish.

### Consent

The researcher or clinical trials nurses will obtain informed consent from all participants in the VOICE Study. All participants will be provided with the relevant Participant Information Statement and Consent Form, according to whether are participating at the intervention or control site and will be required to read and understand the contents of this document and provide their consent via a signature before they are enrolled in the study.

### Confidentiality

Where personal information about study participants is collected, stored, accessed, used, or disposed of, the researcher will ensure that the privacy and confidentiality of the participants is maintained. Any information obtained in connection with this study that could identify a participant will remain confidential. It will only be disclosed with the participant’s permission. In any publication, information will be provided in such a way that participants will not be identified. All computerised data will be entered and stored on a secure hard drive in a de-identified manner, with password access for designated research staff only. Only the researcher and her supervisors will have access to this data.

### Access to data

At the completion of the study, all data will be stored in archive boxes, labelled and placed in a locked storage room, which will be protected by a coded keypad. All data and information associated with this research study will be kept securely for 7 years according to NH&MRC requirements [33]. The data collected will form part of a formal handover by the researcher at the completion of the study.

### Dissemination policy

The results of the VOICE Study will be disseminated in appropriate peer reviewed journals. A report will also be provided to participating family members who have requested a summary of the research findings. Presentations of the results to the participating sites will also be undertaken.

## Discussion

Palliative care patients are generally seen as a vulnerable group in terms of research because of their limited life expectancy and the impost on them to participate in research when they may be compromised physically and psychosocially. Nevertheless, the currently limited high-level evidence to support the routine use of family meetings in the inpatient palliative care setting indicates that this feasibility study is warranted. In particular, there is limited evidence for patient and family benefits using validated outcomes measures and limited qualitative evidence to demonstrate advantages for clinicians.

This study will determine whether planned Patient-Centred Family Meetings are feasible and acceptable to participating patients, families and clinicians. It will also assess the suitability and feasibility of the selected outcome measures for patients and families. In addition, the study is expected to enhance the understanding of the benefits and burdens of participating in a planned Patient-Centred Family Meetings for patients, families and clinicians. The results will also inform the need for a possible Phase III Randomised Controlled Trial.
